# Is the population of *Crotalus durissus* (Serpentes, Viperidae) expanding in Brazil?

**DOI:** 10.1186/1678-9199-19-30

**Published:** 2013-12-05

**Authors:** Marcelo Ribeiro Duarte, Frederico Alcântara Menezes

**Affiliations:** 1 Laboratory of Zoology Collection, Butantan Institute, Av. Vital Brazil, 1500 São Paulo, CEP: 05503-900, São Paulo State, Brazil

**Keywords:** Rattlesnake, Habitat degradation, Species conservation, Animal distribution

## Abstract

*Crotalus durissus* are found from Mexico to northern Argentina in a highly disjunct distribution. According to some studies, this species is prone to occupy areas disturbed by human activities and floods comprise a plausible method of dispersal as inferred for some North American rattlesnakes. Based on the literature, it seems plausible that *Crotalus durissus* expanded their natural distribution in Brazil due to floods, but only in a few municipalities in Rio de Janeiro State. Data entries of Butantan Institute, in São Paulo, Brazil, from 1998 to 2012 show a declining tendency of snakes brought by donors. In addition, research shows no evidence of *Crotalus durissus* being an expanding species in the Brazilian territory.

## Findings

It seems unquestionable that the wide-ranging *Crotalus durissus* complex is derived from a North American ancestor that spread towards South America [1]. These snakes are found in seasonally dry formations from Mexico to northern Argentina, but are absent from the Central American and Amazonian rainforests [1]. This highly discontinuous distribution includes open habitats both north and south of the Amazon rainforest as well as isolated open formations within it, avoiding the forest itself [2]. *Crotalus durissus* is likely to be found in areas disturbed by human activities and takes advantage of the effects of deforestation in Atlantic rainforest regions [3-5].

**Figure 1:**
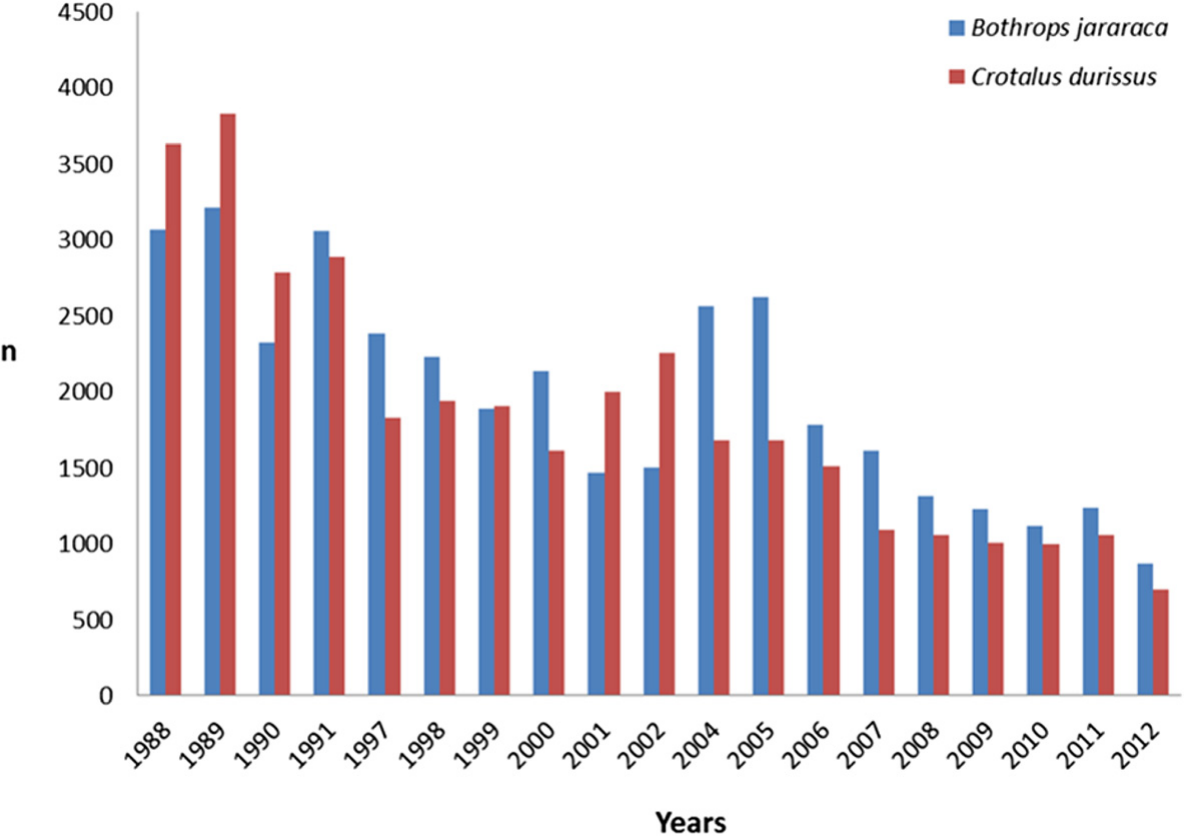
**Entry of *Bothrops jararaca *** **and *Crotalus durissus *** **(Serpentes, Viperidae) per year at Butantan Institute, São Paulo, SP, Brazil.**

According to Klauber [6], although floods are an important method of dispersal of rattlesnakes, after severe ones, *Sistrurus catenatus* population tends to decrease due to reduced prey availability [7]. Indeed, this natural transport was considered the dispersal mode for Brazilian rattlesnakes in some localities of Rio de Janeiro State during the 1950s [8]. In addition, Atlantic forest fragmentation has been suggested as responsible for *Crotalus durissus* territorial expansion and supposed increased density [4]. Bastos *et al.*[8] proposed that this picture may represent the recent invasion of the species into disturbed areas of the Atlantic forest. In this brief correspondence, we examine the growing reputation of *Crotalus durissus* as an expanding species in Brazil, highlighting the implications for land use, distribution, conservation, and epidemiology since this species is responsible for 7.7% of the 20.000 human snakebites that occur in Brazil annually [9]. Entry records of *Crotalus durissus* specimens from the whole Brazilian territory at Butantan Institute (IBSP), a traditional research center, between 1988 and 1990 were compared with those of 1991, 1997–2012 (data from 2003 missing), and statistically analyzed by Past® software (Figure 1) [4].

The gap between 1992 and 1996 is a result of lack of data in the annual reports of Butantan Institute. Records of *Crotalus durissus* specimens found in the Rio de Janeiro State in the Museum of Zoology of the University of São Paulo – MZUSP (SP state), Vital Brazil Institute – IVB (RJ state), National Museum of Rio de Janeiro – MNRJ (RJ state), and IBSP are listed in Appendix.

Since the ratio of *Crotalus durissus* received by IBSP was considered greater than that of *Bothrops jararaca* by Sazima [4], and in order to compare and statistically confirm such tendency, we analyzed the entry of both species in the following periods: 1988–1990, 1991, and 1997–2012.

Regression analysis showed decreasing values for both species in the period (R^2^ = 0.89 and 0.70 respectively, and p = 0.0001). Analysis of covariance showed no difference among species in number of entries, regardless of the year, as well as in the decreasing rate among species during the period (F = 3.87, p = 0.0573). *Crotalus durissus* has no voucher specimens from Rio de Janeiro State in MZUSP, and only one specimen is available outside Brazil at the American Museum of Natural History – AMNH, New York, USA [10]. Although lacking some specimens to be added to their herpetological collections, IVB and MNRJ are together harboring sixty specimens of *Crotalus durissus* from Rio de Janeiro state. These records began in 1988 in IVB and in 2007 in MNRJ.

## Closing remarks

Species face the challenge of living and coexist in complex landscape mosaics that include original habitats, new environments, and urban and agricultural zones [11,12]. Regardless of the motivation, the desire to conserve reptiles and to better understand their ecology requires knowledge of their status, distribution, and factors that contribute to the decline or increase of their population [13].

It appears to be consensual that *Crotalus durissus* is prone to occupy areas disturbed by human activities [3,4,8,14,15]. However, the suitability of *Crotalus durissus* for cleared areas and the edge effects on performance and population dynamics is based only on the authors’ personal experience. In fact, there is substantial discrepancy among recent studies about the existence and intensity of edge effects [16].

According to Tozetti and Martins [15], the combination of shaded and exposed substrate could increase the thermoregulatory possibilities for rattlesnakes in Atlantic forest areas transformed by human activity due to the species low thermal selectivity. However, supposed expansive propensities are counterbalanced by several underrated limiting processes (e.g. inadequate shelter and microclimate, competition, prey availability, ants; predators, parasites, and diseases) in a mosaic landscape [4,14].

If intensive agricultural practices are considered, a fair analysis would have to consider other aspects in the equation, since important levels of disturbance factors like machinery displacement, fire, exposition to soil corrective and supplementary nutrients as well as pesticides must be accounted. In fact, stressor factors such as habitat degradation and human predation affect snake population as well as unrestrained development, pollution, unwise land use practices and contaminants [13,17,18].

Conversion of natural fragments into agricultural fields *versus* snake survival is a doubtful matter [19]. Landscape-scale disturbances, such as grazing, fire, residential development, agriculture, and forestry, occur throughout the habitat of *Crotalus* from North America to Uruguay and Brazil [20,21]. Only a few crotalines receive special protection and even fewer have been subjected to detailed assessment of their conservation status [22].

From the epidemiological viewpoint, the statement that the historic distribution of *Crotalus durissus* does not include Rio de Janeiro state until the 1990s is not true [23]. In fact, there are voucher specimens from this state (see Appendix) from the municipalities of Niterói (AMNH 27731: 1940) and Miracema (IBSP 11265, 11266: 1947) [10]. Some evidence of this involuntary colonist in Rio de Janeiro state could be based on the lack of epidemiological records [24-26]. Additionally, it is reasonable to believe in the hypothesis of natural transport by floods; since it is highly unlikely that such a large snake would have gone unnoticed [8,27].

Typical limitations of studies related to the effects of habitat change on tropical herpetofauna include the lack of reliable baseline [28]. Moreover, we suggested caution against the claim that cleared areas *per se* will provide *Crotalus durissus* adaptation improvement. To conserve herpetofauna in urban areas (or in degraded landscape) structural complexity in remnant habitat patches must be maintained [29].

At least for rattlesnake species from North America, Uruguay, Amazonian savanna enclaves, and Aruba Island the scenario is not promising [17,20,21,30-35]. The perspective that rattlesnakes are expanding their territory in Brazil creates a false perception that this animal does not deserve concern or even protection. Clearly, careful studies taking into account the above-mentioned variables are essential to document temporal changes in rattlesnake populations in Brazil. To the best of our knowledge, such research has not been carried out yet.

## Appendix

### Vouchers of *Crotalus durissus* from Rio de Janeiro state, Brazil

**AMNH** Niterói: 27731.

**IBSP** Barra Mansa: 17144, 54911, 55712; Barra do Piraí: 40055, 48087; Miracema: 11265, 11266, 29290; Paraíba do Sul: 54665, 54731; Paraty: 31818; Resende: 67981; Rio das Flores: 60650; Três Rios: one record without voucher (February, 5, 1998); Valença: 26718, 51418, 51426, 51797, 52877, 52878, 52879, 55531, 55532, 55533, 55534.

**IVB** Areal: 2772; Itatiaia: 645; Paraíba do Sul: 1390, 1409, 1577, 2207; Três Rios: 2763, Valença (1990–1996): 1658, 1665, 1698, 1869, 1954, 1955, 1986, 2070, 2090, 2102, 2103, 2107, 2108, 2157, 2175, 2201, 2249, 2412, 2419, 2420, 2428, 2429, 2430, 2431, 2433, 2450, 2459, 2462, 2501, 2509, 2691, 2713, 2778, 2877, 2920*; Volta Redonda: 1502.

**MNRJ**: BR 040 km 797: 20792; Rio das Flores: 16976–79, 16974–75, 18173; Valença: 19495–97.

**MZUSP**: No records.

*According to Bastos [8], IVB received 82 specimens from this locality between 1999–2003.

## Abbreviations

AMNH: American museum of natural history, New York, USA; IBSP: Herpetological collection “Alphonse Richard Hoge”, Butantan Institute, São Paulo, SP, Brazil; IVB: Vital Brazil Institute, Niterói, RJ, Brazil; MNRJ: National Museum, Rio de Janeiro, RJ, Brazil; MZUSP: Museum of Zoology of the University of São Paulo, São Paulo, SP, Brazil.

## Authors’ contributions

MRD participate in the design of the study and drafted the manuscript. FAM carried out the data acquisition and statistical analysis. Both authors read and approved the final manuscript.
